# Supplementary material for: Understanding Glioblastoma Dynamics Using 3D Organoids and Engineered Extracellular Matrix
Source: Adv Sci (Weinh). 2026 Mar 28;13(24):e22926. doi: 10.1002/advs.202522926 (PMC13115983; doi:10.1002/advs.202522926)
Supplement: Supplementary file 1 — Supporting File: advs74447‐sup‐0001‐SuppMat.pdf. [file ADVS-13-e22926-s001.pdf]

Supplementary Information

Understanding Glioblastoma Dynamics Using 3D Organoids and Engineered Extracellular Matrix

Autumn McManis[1,2,3], Charles Ashley Jimenez[2], Abha Shirolkar[2], Syed Raza ur Rehman[3], Sumana Mallick[2,3], Malea Murphy[4], Akhilesh K Gaharwar[1,3,5], and Irtisha Singh[1,2,3*]

[1]Interdisciplinary Graduate Program in Genetics and Genomics, Texas A&M University, College Station, Texas 77843

[2]Department of Cell Biology and Genetics, Texas A&M Health Science Center, Bryan, Texas 77807

[3]Department of Biomedical Engineering, Texas A&M University, College Station, Texas 77843

[4]Department Medical Physiology, Texas A&M Health Science Center, Bryan, TX 77807

[5]Department of Materials Science and Engineering, Texas A&M University, College Station, Texas 77843

*Corresponding author: isingh@tamu.edu (Dr. Irtisha Singh)

Supplementary Table S1. Gene expression levels from Mack, et al, *Chromatin landscapes reveal developmentally encoded transcriptional states that define human glioblastoma*, 2019[1]. Gene expression levels in Fragments Per Kilobase of transcript per Million mapped reads (FPKM) of GSCs. Reference names from the 2019 paper in parentheses after nomenclature used in this study. Selected results are markers for proneural GSCs (OLIG2, BCAN), mesenchymal GSCs (STAT3, RUNX2), angiogenesis (VEGFA, FGF2), stemness (CD133), proliferation (Ki67), hypoxia (CAIX), astrocytic differentiation (GFAP), oligodendrocytic differentiation (MOG), pericytic differentiation (CD248), and endothelial differentiation (CD31).

GENE Symbol	pGSC (GSC1)	mGSC (GSC17)
OLIG2	35.56151990	0.04018502
BCAN	12.82729650	2.07809460
STAT3	44.98286630	101.00168300
RUNX2	0.01833006	4.81458184
VEGFA	49.95237240	19.14081730
FGF2	4.68251591	2.20753411
CD133 (PROM1)	2.31047632	19.56750560
Ki67 (MKI67)	7.74293101	17.65308010
CAIX (CA9)	0.63039461	0.18397750
GFAP	8.97649805	0.38562261
MOG	0	0.00892049
CD248	0	0.06870684
CD31 (PECAM1)	0	0.36166517

Supplementary Table S2. Fluorescence image thumbnails used in this publication. For each figure, the presence or absence of fluorescence images is indicated (N/A where not applicable). For figures containing fluorescence data, the table specifies whether thumbnails were generated as maximum-intensity projections (MIP) of z-stacks or as MIPs with uniform brightness and contrast adjustments applied for visualization.

<table>
<tr><th colspan="2">Fluorescent Image Thumbnails Used in Publication</th></tr>
<tr><th>MIP (unadjusted)</th><th>MIP (brightness/contrast adjusted)</th></tr>
<tr><td colspan="2">Figure 1
N/A</td></tr>
<tr><td colspan="2">Figure 2</td></tr>
<tr><td>all fluorescence panels</td><td>none</td></tr>
<tr><td colspan="2">Figure 3</td></tr>
<tr><td>GFAP panel</td><td>MOG, CD248, CD31 panels</td></tr>
<tr><td colspan="2">Figure 4
N/A</td></tr>
<tr><td colspan="2">Figure 5</td></tr>
<tr><td>Panel B</td><td>Panel C</td></tr>
<tr><td colspan="2">Figure 6</td></tr>
<tr><td>none</td><td>all fluorescence panels</td></tr>
<tr><td colspan="2">Figure S1
N/A</td></tr>
<tr><td colspan="2">Figure S2</td></tr>
<tr><td>none</td><td>all fluorescence panels</td></tr>
</table>

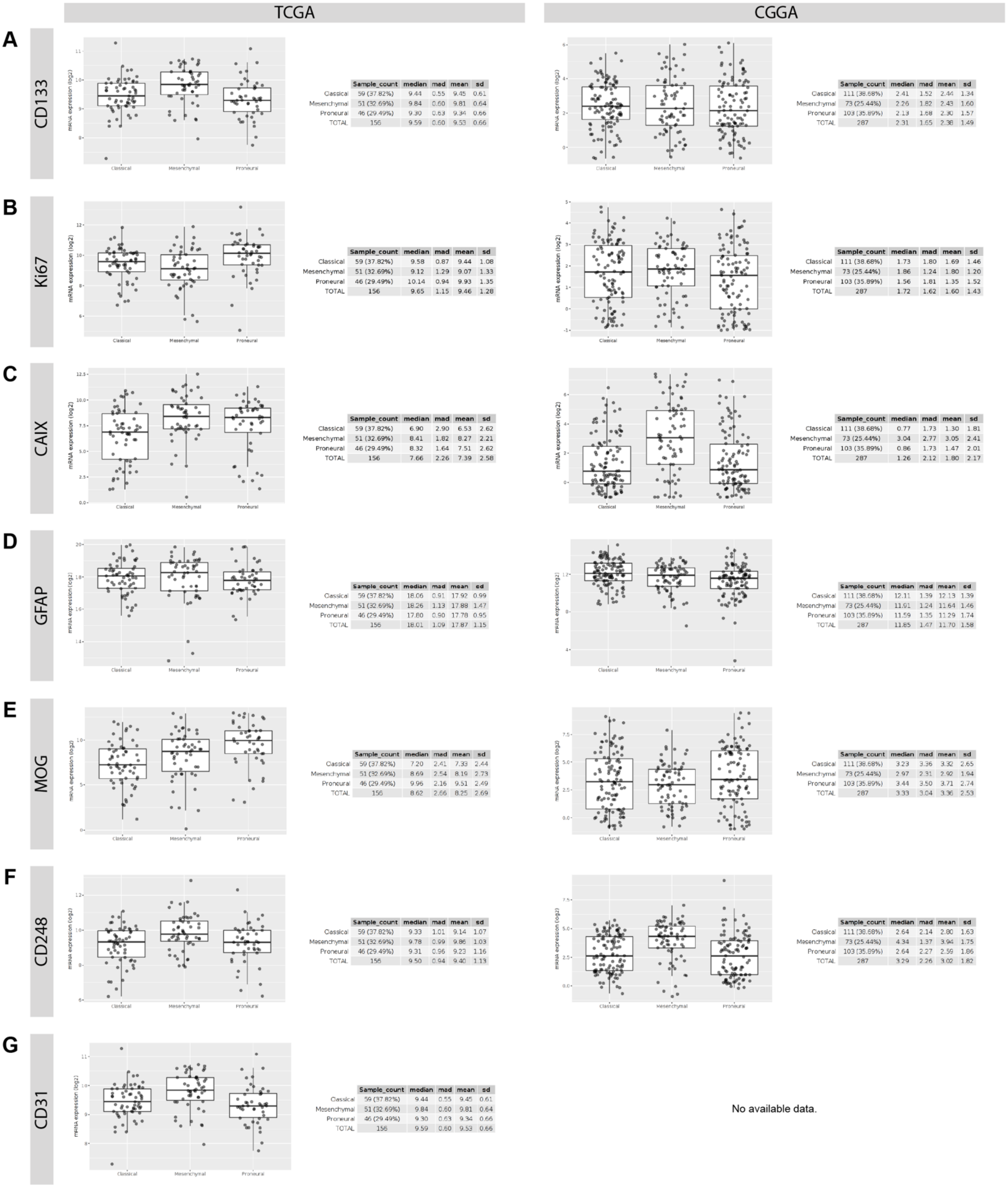

	Sample_count	median	mad	mean	sd
Classical	59 (37.82%)	9.44	0.55	9.45	0.61
Mesenchymal	51 (32.69%)	9.84	0.60	9.81	0.64
Proneural	46 (29.49%)	9.30	0.63	9.34	0.66
TOTAL	156	9.59	0.60	9.53	0.66

	Sample_count	median	mad	mean	sd
Classical	111 (38.68%)	2.41	1.52	2.44	1.34
Mesenchymal	73 (25.44%)	2.26	1.82	2.43	1.60
Proneural	103 (35.89%)	2.13	1.68	2.30	1.57
TOTAL	287	2.31	1.65	2.38	1.49

	Sample_count	median	mad	mean	sd
Classical	59 (37.82%)	9.58	0.87	9.44	1.08
Mesenchymal	51 (32.69%)	9.12	1.29	9.07	1.33
Proneural	46 (29.49%)	10.14	0.94	9.93	1.35
TOTAL	156	9.65	1.15	9.46	1.28

	Sample_count	median	mad	mean	sd
Classical	111 (38.68%)	1.73	1.80	1.69	1.46
Mesenchymal	73 (25.44%)	1.86	1.24	1.80	1.20
Proneural	103 (35.89%)	1.56	1.81	1.35	1.52
TOTAL	287	1.72	1.62	1.60	1.43

	Sample_count	median	mad	mean	sd
Classical	59 (37.82%)	6.90	2.90	6.53	2.62
Mesenchymal	51 (32.69%)	8.41	1.82	8.27	2.21
Proneural	46 (29.49%)	8.32	1.64	7.51	2.62
TOTAL	156	7.66	2.26	7.39	2.58

	Sample_count	median	mad	mean	sd
Classical	111 (38.68%)	0.77	1.73	1.30	1.81
Mesenchymal	73 (25.44%)	3.04	2.77	3.05	2.41
Proneural	103 (35.89%)	0.86	1.73	1.47	2.01
TOTAL	287	1.26	2.12	1.80	2.17

	Sample_count	median	mad	mean	sd
Classical	59 (37.82%)	18.06	0.91	17.92	0.99
Mesenchymal	51 (32.69%)	18.26	1.13	17.88	1.47
Proneural	46 (29.49%)	17.80	0.90	17.78	0.95
TOTAL	156	18.01	1.09	17.87	1.15

	Sample_count	median	mad	mean	sd
Classical	111 (38.68%)	12.11	1.39	12.13	1.39
Mesenchymal	73 (25.44%)	11.91	1.24	11.64	1.46
Proneural	103 (35.89%)	11.59	1.35	11.29	1.74
TOTAL	287	11.85	1.47	11.70	1.58

	Sample_count	median	mad	mean	sd
Classical	59 (37.82%)	7.20	2.41	7.33	2.44
Mesenchymal	51 (32.69%)	8.69	2.54	8.19	2.73
Proneural	46 (29.49%)	9.96	2.16	9.51	2.49
TOTAL	156	8.62	2.66	8.25	2.69

	Sample_count	median	mad	mean	sd
Classical	111 (38.68%)	3.23	3.36	3.32	2.65
Mesenchymal	73 (25.44%)	2.97	2.31	2.92	1.94
Proneural	103 (35.89%)	3.44	3.50	3.71	2.74
TOTAL	287	3.33	3.04	3.36	2.53

	Sample_count	median	mad	mean	sd
Classical	59 (37.82%)	9.33	1.01	9.14	1.07
Mesenchymal	51 (32.69%)	9.78	0.99	9.86	1.03
Proneural	46 (29.49%)	9.31	0.96	9.23	1.16
TOTAL	156	9.50	0.94	9.40	1.13

	Sample_count	median	mad	mean	sd
Classical	111 (38.68%)	2.64	2.14	2.80	1.63
Mesenchymal	73 (25.44%)	4.34	1.37	3.94	1.75
Proneural	103 (35.89%)	2.64	2.27	2.59	1.86
TOTAL	287	3.29	2.26	3.02	1.82

	Sample_count	median	mad	mean	sd
Classical	59 (37.82%)	9.44	0.55	9.45	0.61
Mesenchymal	51 (32.69%)	9.84	0.60	9.81	0.64
Proneural	46 (29.49%)	9.30	0.63	9.34	0.66
TOTAL	156	9.59	0.60	9.53	0.66

Supplementary Figure S1. Subtype specific expression patterns from TCGA and CGGA databases. (A-G) RNAseq expression patterns seen in classical, proneural, or mesenchymal type GBM. Results reflect GSC health (A-C) and differentiation markers (D-G). This figure is generated using GlioVis (Bowman et al., Neuro Oncol 2017)[2] (https://gliovis.bioinfo.cnio.es/)

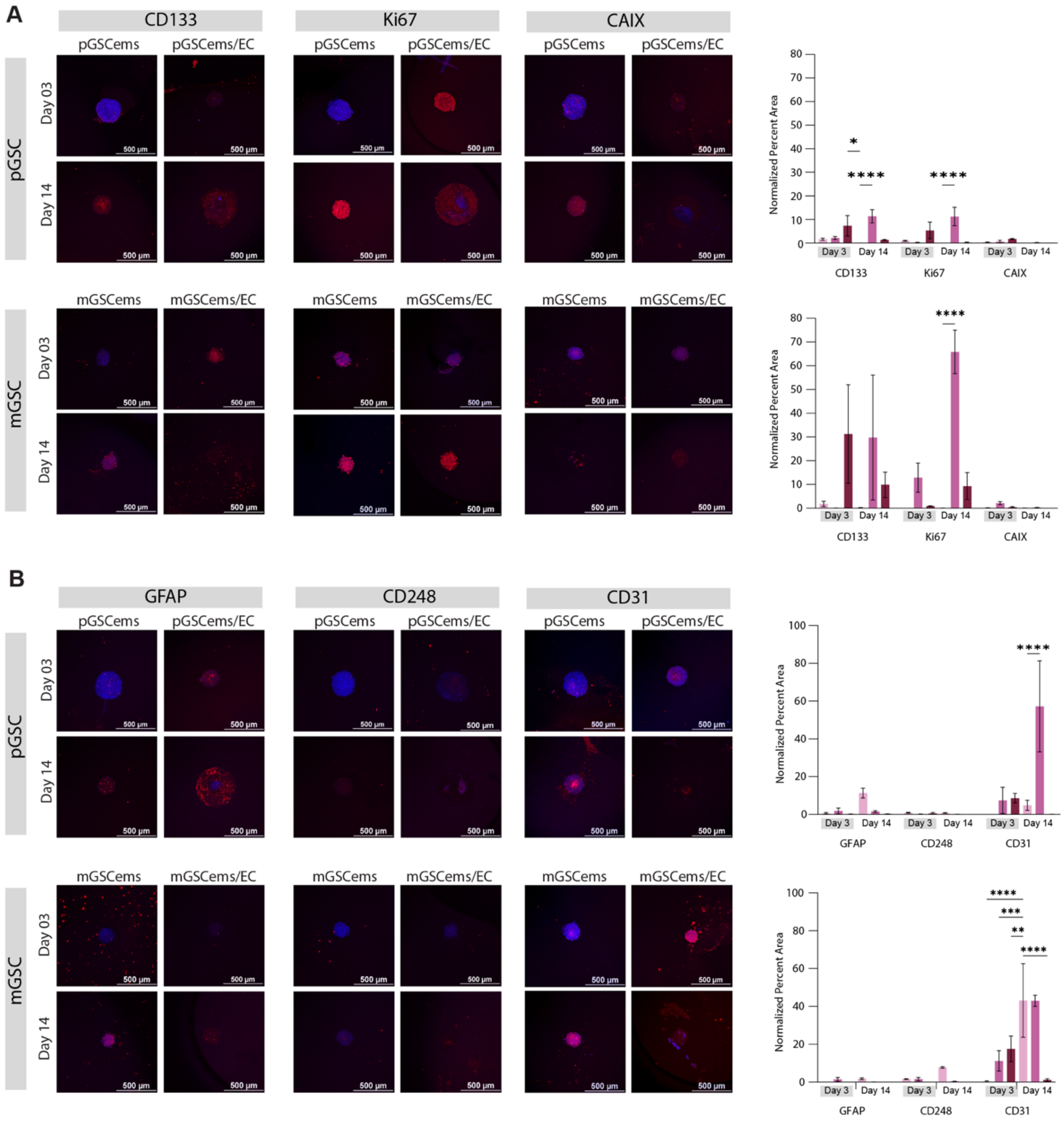

Supplementary Figure S2. Expanded IF staining results seen in Figure 6. (A) Immunofluorescence (IF) staining and quantification of stemness (CD133), proliferation (Ki67), and hypoxia (CAIX) markers in pGSC and mGSC spheroids under encapsulated and co-culture conditions. (B) IF staining and quantification of astrocytic (GFAP), pericytic (CD248), and endothelial (CD31) differentiation markers, highlighting enhanced lineage specification with EC co-culture. Matrigel Spheroid percent areas previously presented have been included in the graphs for reference. Data are presented as mean ± SD (N = 3) with the following exceptions due to sample loss: pGSCemsec-d03 (N = 2) and mGSCems-d03 (N = 2). Statistical analysis was performed using two-way ANOVA with Dunnet's post hoc test (*$p \leq 0.05$, **$p \leq 0.005$, ***$p \leq 0.0005$, ****$p \leq 0.00005$). Note: Portions of this figure are presented in Figure 6 of the main manuscript. All images (Ki67 and CD31) and corresponding quantitative graphs are reproduced here for completeness and improved readability.

References

(1) Mack, S. C.; Singh, I.; Wang, X.; Hirsch, R.; Wu, Q.; Villagomez, R.; Bernatchez, J. A.; Zhu, Z.; Gimple, R. C.; Kim, L. J. Y.; et al. Chromatin landscapes reveal developmentally encoded transcriptional states that define human glioblastoma. *Journal of Experimental Medicine* **2019**, *216* (5), 1071–1090. DOI: 10.1084/jem.20190196.

(2) Bowman, R. L.; Wang, Q.; Carro, A.; Verhaak, R. G.; Squatrito, M. GlioVis data portal for visualization and analysis of brain tumor expression datasets. *Neuro Oncol* **2017**, *19* (1), 139-141. DOI: 10.1093/neuonc/now247.
